# Low levels of knowledge and practice of occupational hazards among flower farm workers in southwest Shewa zone, Ethiopia: a cross-sectional analysis

**DOI:** 10.1186/s12889-021-10254-5

**Published:** 2021-01-28

**Authors:** Debela Hinsermu Geleta, Mekuriaw Alemayehu, Geta Asrade, Tesfaye Hambisa Mekonnen

**Affiliations:** 1 The National Regional State of Oromia, Labour and Social Affairs Bureau, Addis Ababa, Ethiopia; 2 Department of Environmental and Occupational Health and Safety, Institute of Public Health, College of Medicine and Health Sciences, University of Gondar, Gondar, Ethiopia; 3 Department of Health Systems and Policy, Institute of Public Health, College of Medicine and Health Sciences, University of Gondar, Gondar, Ethiopia

**Keywords:** Knowledge, Practice, Occupational hazards, Flower farms, Ethiopia

## Abstract

**Background:**

Over the last decade, flower farms have been rapidly growing in Ethiopia. Following the advent and development of the sector, various work-related chemical, biological, physical, psychosocial, and ergonomic hazards have been emerging unacceptably, with increased risks of exposures for workers and local communities. However, evidence that describes knowledge and prevention practice of occupational hazards among flower farm workers in the country is little documented. The knowledge and safety practice of occupational hazards among flower farm workers in Ethiopia were explored in the current study.

**Methods:**

A cross-sectional survey of 471 flower farm workers was implemented from March to April 2017. A stratified random sampling technique was used to select the eligible participants. An interviewer-administered questionnaire was used to collect data, and the data were entered in to Epi Info program version 7 and analyzed by SPSS program version 20. Bivariate and multivariate linear regression analyses were performed to evaluate significance of associations at < 0.05 *p*-values.

**Results:**

A total of 451 flower farm workers were interviewed with a response rate of 95.7%. The majority, 72.1% (*N* = 325) were females. Mean age was 24.1 (SD + 6.5) years. About 39.2% (*N* = 177) of the participants had good knowledge on occupational hazards. The level of safety practice was 26.6% (*N* = 120). The level of knowledge on occupational hazards was affected by level of education [AOR: 20.03;95% CI (16.30,23.75)], work experience [AOR: 5.97; 95% CI (4.22,7.72)], and type of employment [AOR: 5.35; 95% CI (2.50,8.19)], whereas the level of safety practice was influenced by regular use of personal protective equipment (PPE) [AOR:17.53;95% CI (13.36,21.71)], level of knowledge [AOR: 7.29; 95% CI (3.87,10.73)], and provision of appropriate PPE [AOR: 4.59; 95% CI (2.34,8.86)].

**Conclusion:**

This study revealed the levels of knowledge and safety practice towards occupational hazards were low. The knowledge on occupational hazards was significantly affected by the level of education and duration of employment. Moreover, the use of PPE and level of knowledge considerably influenced safety practice. Therefore, we recommend employers to ensure that workplace health and safety programs account for workers’ level of education and work experience. It is also pivotal to provide workers witha suitable PPE and instructions on its use, and to arrange safety communication in the local languages at the relevant workplaces.

**Supplementary Information:**

The online version contains supplementary material available at 10.1186/s12889-021-10254-5.

## Background

In Ethiopia, flower farms have been rising extensively over the last decade [1]. The reasons for the rapid growth of the sectors include favorable climate, government support, proximity to the global market, readily available transportation services, favorable investment policies, and abundant and cheap labor force in the country [2, 3]. The industry has made a significant contribution to the national economy through the export of cut flowers and creation of employment opportunities [4, 5]. Working with the flower farm industries, however, presents several safety and health challenges [1, 5, 6]. For example, hazards associated with the industry premises sucha as chemical, biological, physical, psychosocial, and ergonomic hazards have been emerging unacceptably, with increased risks of exposures for workers and the surrounding communities [7]. Moreover, vulnerable groups of workers, including a large number of young men and women and daily laborers often engage in harsh environmental conditions like excessive heat and cold for long working hours, and they also work with various hazardous chemical pesticides [1, 8, 9].

According to the International Labour Organization (ILO), each year, about 2.3 million workers die because of occupational accidents and diseases, whereas 337 million suffer from it [10]. Flower industries are the major sources of these accidents and diseases due to various occupational and environmental hazards related to them [11]. Working in flower farm industries, therefore, exposes employees to various adverse health outcomes including respiratory, neurological, and dermal symptoms [5, 12–16].

Chemical fertilizers and pesticides are used intensively in Ethiopia, in parallel with the expansions of agricultural sectors [1, 2, 4]. Because health and safety enforcement in Ethiopia is relatively weak, practice to handle and safe procedure to use those chemicalsis often rarely observed. Besides, employees working with the chemicals usually lack knowledge and skill due to shortage of training and awareness on the hazards associated with the chemicals [4]. Further, the use of and access to appropriate personal protective equipment (PPE) in these industries is often limited [5, 15].

Several factors notably influence the knowledge and safety practice of workers on workplace hazards. For example, the level of knowledge on occupational hazards is significantly related to level of education [17]. Knowledge of workers on occupational hazards is also affected by safety trainings and job tenure [18]. Similarly, the level of safety practice is influenced by socio demographic factors including age [17]. Safety practice has also a remarkable relation with the level of knowledge [5, 19].

To date, the health and working conditions of workers [4, 5, 14] and the environmental impacts of flower farms in Ethiopia have been discussed [15, 20]. However, there is a scarce research and minimal information on the knowledge and practice of occupational hazards. Therefore, this study investigated the knowledge and practices of occupational hazards as well as their associated factors among flower farm workers in Ethiopia. Exploring the diverse factors that determine the levels of knowledge and prevention and control practices of occupational hazards is central to public health programs.

## Methods

### Study design

Flower farms based cross-sectional survey.

### Study area and period

This study was conducted in Southwest Shewa zone, National Regional State of Oromia, Ethiopia, from March to April 2017. Southwest Shewa zone is one of the zones of the National Regional State of Oromia, Central Ethiopia. Wolliso, the capital of the zone, is an ideal place for investment activities, particularly agroindustry. The town is 114 km from Addis Ababa, the capital of Ethiopia, and it has 12 districts and town administration. At the time of data collection, there were over 25 industries in the area, of which five were flower farms. Moreover, during data collection period, there were about 1500 employees working in the flower farm industries.

### Populations

All workers in the flower farms in Southwest Shewa zone were the source population, whereas those who met the inclusion criteria and available during data collection were the study population.

### Inclusion and exclusion criteria

Employees who had worked 3 months and above prior to the data collection were included, while those who were sick, on annual and maternity leaves were excluded.

### Sample size determination and sampling procedures

Epinfo program version 7 was used to calculate the required sample. We derived the levels of knowledge and safety practice of occupational hazards from previous studies [6, 21, 22]. Accordingly, proportions of 72.9 and 76.3% with a Confidence Interval (CI) of 95 and 5% margins of error were presumed and 268 and 248 samples were calculated for the levels of knowledge and safety practice, respectively. Similarly, for each specific objective, three associated factors including training on workplace conditions (43.1% prevalence and 3.5 Odds Ratio (OR) with a CI of 95%), not using PPE (30.2% prevalence and 4.065 OR with a CI of 95%), and not using shower (43% prevalence and OR 0.569 with a CI of 95%) were taken from the aforementioned studies to attain 118, 136 and 428 samples, respectively. Finally, we took the largest sample size (428) to ensure the adequacy of the sample for statistical power. We assumed a 10% for none response rates which gave the final sample of 471.

The stratified sampling technique was employed, considering that the populations in each selected industry were heterogeneous. The five floriculture industries were included purposively to attain the required sample. A proportional allocation was used to derive sample from each stratum. Finally, using the lists of workers’ identification numbers provided them by the industries, we applied a computer-generated random number to reach each participant.

### Data collection tools and quality assurance

Data were collected by use of a structured and interviewer-administered questionnaire. We developed the questionnaire after a meticulous review of published works [17, 23–27]. The questionnaire has four sections. The first section contains socio-demographic characteristics including sex, age, educational status, profession, marital status, and work experience. The second, included questions related to the level of knowledge on occupational hazards. The third and fourth sections provided detailed information on safety practices and factors affecting the levels of knowledge and safety practice (dependent variables), respectively. To assess the level of knowledge, detailed lists of knowledge questions (14 items such as knowledge on safety communication (safety labels, symbols, pictograms, guidelines), material safety data sheets (MSDS), healthy effects of hazards, and routes of exposures) were presented. Responses to the questions are coded such that correct answers (Yes) scored one and incorrect (No) zero. Knowledge score was then categorized as not knowledgeable for < 50% and knowledgeable for > 50% correct responses from the overall scores [27].

A questionnaire containing 16 items was administered to assess the level of safety practice and the scores were categorized as poor for < 50% and good practice for > 50% correct (Yes) responses out of the total scoring [26]. The questionnaire was first prepared in English and translated to Afan Oromo (the local language) and retranslated to English by independent language experts to verify its consistency. Finally, the Afan Oromo version of the questionnaire was interviewer-administered to the participants at their work sites. Moreover, we designed a standard checklist to evaluate workplace hazards and observe employees’ onsite safety practice.

To ensure the quality of data, first, a pretest was conducted on 5% of the sample (48 workers) in flower farm at Sebeta flower farm, which have similar characteristics as those included in the final survey. Based on the test results, we reduced the number of questions (without changing what was intended to be measured) to minimize the time needed for interviews, and we modified ambiguous questions. Secondly, training and orientation was given to data collectors (2 females, 4 males) and two supervisors on issues relating to the objectives of the study, confidentiality of data, consent and appropriate time for data collection.

### Data processing and analysis

The collected data were entered into Epi-info version 7 tocleanand code the collected data. Double entry was performed with 10% data to verify errors that couldoccur during the entireentry process. The data were analyzed with SPSS program version 20. The findings were presented using descriptive statistics includingfrequency tables, graphs, percentages, means with standard deviations. Knowledge and practice questions were marked with ‘0’ for ‘No ‘and ‘1’ ‘Yes’ response.

We checked the reliability of the knowledge and practice items using a Cronbach’s Alpha Coefficient. As such, the knowledge questions yielded a Coefficient of 0.78, while that of the practice 0.81. It was previously shown that a Cronbach’s Alpha Coefficient of a given instrument is considered reliable if it is > 0.65. Before running the multivariable linear regression analysis in the final model, linearity, normality, outliers, autocorrelation, multicollinearity and independence of errors/residues of the variables were also examined. The multicollinearity test was done using variable inflation factor (VIF) and all variable**s** showed VIF < 5. A bivariate linear regression analysis was performed to examine associations of each independent variable and knowledge and practice separately. Independent variables with < 0.2 *p*-values in this type of analysis were exported to the multivariable linear regression analysis to control effects of potential confounders. We set the significance of associations at < 0.05 *p*-values, while adjusted odds ratio (AOR) with 95% confidence interval (CI) was used to determine the strength of associations.

## Results

### Socio-demographic characteristics of the respondents

A total of 451 flower farm workers were interviewed with a response rate of 95.7%. Seventy-two percent (*N* = 325) of those surveyed participants were females. Mean age of the participants was 24.1 (SD + 6.5) years. Of the interviewees, 86.3% (*N* = 389) of them were Oromo, 8.0% (*N* = 36) Gurage, and 5.7% (*N* = 26) Amhara. The majority, 54.8% (*N* = 247) of the participants were urban, while 45.2% (*N* = 204) were rural residents. More than half, 46.8% (*N* = 211) of the interviewees were temporary and the remaining were permanent workers. With respect to monthly salary, 58.9% (*N* = 266) of the workers earned less than 1500 ETB (50 USD) per month. More than half, 58.1% (*N* = 262) worked in closed and hot conditions, whereas 22.4% (*N* = 101) of the participants worked in open and hot environments **(**Table 1).

**Table 1:** Socio-demographic characteristics of flower farm workers, Ethiopia, 2017

Characteristics (***N*** = 451)	Frequency (N)	Percentage (%)
**Sex**		
Male	126	27.9
Female	325	72.1
**Age**		
< 20 years	170	37.7
20–29 years	203	45.0
> 30 years	78	17.3
**Monthly salary/month**		
< 50 USD	266	59.0
> 50 USD	185	41.0
**Educational level**		
No formal Education	169	37.5
Primary (1–6 grades)	117	25.9
Secondary (7–12 grades)	118	26.2
Diploma and above	47	10.4
**Marital status**		
Single	155	34.4
Married	296	65.6
**Work experience**		
< 2 years	210	46.6
> 2 years	241	53.4
**Working hours per day**		
< 8 h	437	96.9
> 8 h	14	3.1

**Keys**: *N* Number, *USD* United States of America Dollar (1 USD = 30 Ethiopian Birr)

### Organizational and behavioral characteristics

**Fig. 1:**
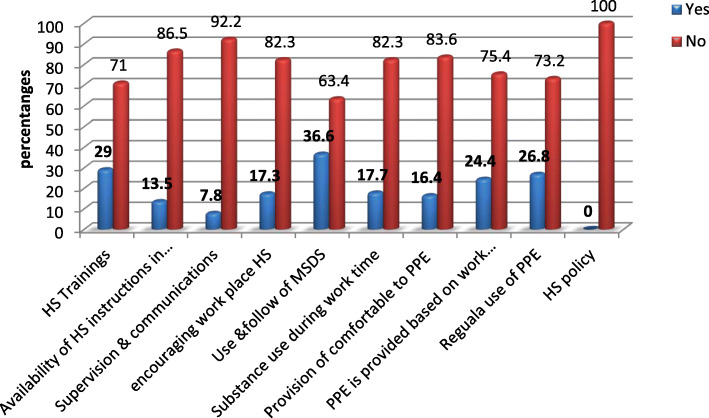
Organizational and behavioral characteristics of the participants

Figure 1 shows that almost all, 92.2% (*N* = 416) of the respondents had no regular supervisions and communication on safe work procedures; 86.5% (*N* = 390) worked without health and safety instructions, symbols and pictograms; 82.7% (*N* = 374) reported there were no programs for health and safety at work.

### Level of knowledge on occupational hazards

Of the participants, 39.2% (*N* = 177) [95% CI (34.8, 43.9)] showed that they had a good level of knowledge (knowledgeable), whereas 60.8% (*N* = 274) [95% CI (56.1, 65.2)] of the participants scored poor (not knowledgeable) on occupational hazards. The mean knowledge score was 7.15 (SD + 1.896). Of the participants, 50.6% (*N* = 228) revealed they had no information about the hazards associated with their current jobs. A lower proportion, 38.6% (*N* = 174) of the sampled workers indicated that their sources of information about occupational hazards was experienced co-workers; 25.1% (*N* = 97) guidelines provided by the manufacturer, and 24.8% (*N* = 112) trainings. Out of the respondents, about 42.4% (*N* = 191) pointed out they knew the presence of chemical hazards, while 36.8% (*N* = 166) and 26.6% (*N* = 120) of them knew the presence of physical and psychosocial hazards, respectively. Table 2 depicts knowledge responses of the participants towards occupational hazards.

**Table 2:** knowledge related responses of the participants, Ethiopia, 2017 (*N* = 451)

Items	Frequency (N)	Percentage (%)
Ever heard occupational hazards in the flower farm		
No	228	50.6
Yes	223	49.4
know occupational hazards (OH) associated with your work	252	55.9
No	199	44.1
Yes		
know nature & conditions of work activities performed in the farm		
No	198	43.9
Yes	253	56.1
Know about forms of occupational hazards		
No	217	48.1
Yes	243	51.9
know the six types of work place hazards		
No	137	30.4
Yes	314	69.6
Know laws regarding OHS in the flower farm		
No	226	50.1
Yes	225	49.9
Know safety communication		
No	245	54.3
Yes	206	45.7
Know about routes of exposure of work place hazards		
No	249	55.2
Yes	202	44.8
Know the major routes of exposures		
No	149	33.0
Yes	302	67.0
know the possible health problems arising from the existing hazards		
No	260	57.6
Yes	191	42.4
Know that workplace hazards can cause environmental pollutions		
No	224	49.7
Yes	227	50.3
know your rights and responsibilities in accident reduction/prevention strategy		
No	286	63.4
Yes	165	36.6
Know about emergency response and preparedness for the control of work place hazards		
No	285	63.2
Yes	166	36.8
Knowing the use of emergency measures in time of accidence occurrence		
No	297	65.9
Yes	154	34.1

### The level of safety practice relating to occupational hazards

**Fig. 2:**
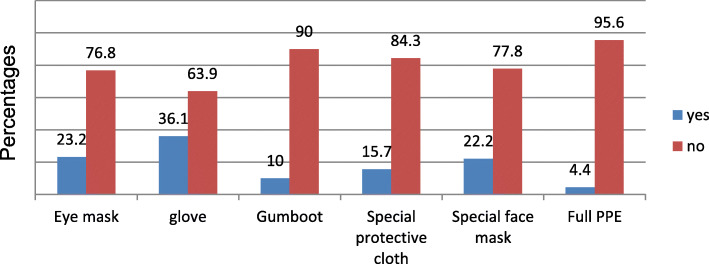
The types of PPE used byworkers

The overall level of safety practice was 26.6% (*N* = 120). About 68.3% (*N* = 308) of the respondents explained they correctly used health and safety communication; 57.2% (*N* = 258) reported sudden occurrences/events; 53.2% (*N* = 240) properly used information and work materials provided by the industries and 53.0% (*N* = 239) of the surveyed workers stated that they used health and safety instructions**.** Regarding the use of personal protective equipment (PPE), 60.1% (*N* = 271) of the respondents indicated they did not use it regularly. Few workers, 4.4% (*N* = 20) observed they used full body protection, while 36.1% (*N* = 163) reported they used gloves (Fig. 2). Moreover, 61.4% (*N* = 277) of the participants reported that waste emitted from the flower farms was not properly disposed; 60.8% (*N* = 274) of the workers did things not recommended such as eating, drinking, not washing hands after work, smoking, taking and washing PPE at home, used empty chemical, fertilizer and other raw material containers for water storage at home and selling. Table 3 describes practice responses of the participants towards occupational hazards.

**Table 3:** Practice related responses of the participants (*N* = 451), Ethiopia, 2017

Items	Frequency (N)	Percentage (%)
Use of health and safety instructions		
No	212	47.0
Yes	239	53.0
Regular use of safe procedures		
No	265	58.8
Yes	186	41.2
Experience of immediate reporting of sudden occurrences/events		
Not reported	193	42.8
Reported	258	57.2
Proper use of instructions and consultations given by owners		
No	293	65.0
Yes	158	35.0
Regular use of PPE		
No	271	60.1
Yes	180	40.9
Appropriate use of available PPE		
No	262	58.1
Yes	189	41.9
Feel comfortable with the use of PPE		
No	383	84.9
Yes	68	15.1
Often use information on material safety data sheet (MSDS)		
No	309	68.9
Yes	142	31.5
Proper use of information and working materials provided by the owners		
No	211	46.8
Yes	240	53.2
Considering wind direction when spraying chemicals and herbicides		
No	329	72.9
Yes	122	27.1
Proper use of first aid services & other emergency measures available during incidence occurrences		
No	324	71.8
Yes	127	28.2
Proper use of accident prevention & control methods available		
No	339	75.2
Yes	112	24.8
Follow safe disposal of wastes		
No	217	48.1
Yes	234	51.2
Use of proper disposal mechanism with the types of wastes emitted from farm		
No	277	61.4
Yes	174	38.6
Never use of empty containers of raw materials for other use		
No	255	56.5
Yes	196	43.5
Never doing unwanted (un-recommended) activities while working		
No	274	60.8
Yes	177	39.2

Keys: *MSDS* Material safety data sheets

### Findings of workplace observations

**Fig. 3:**
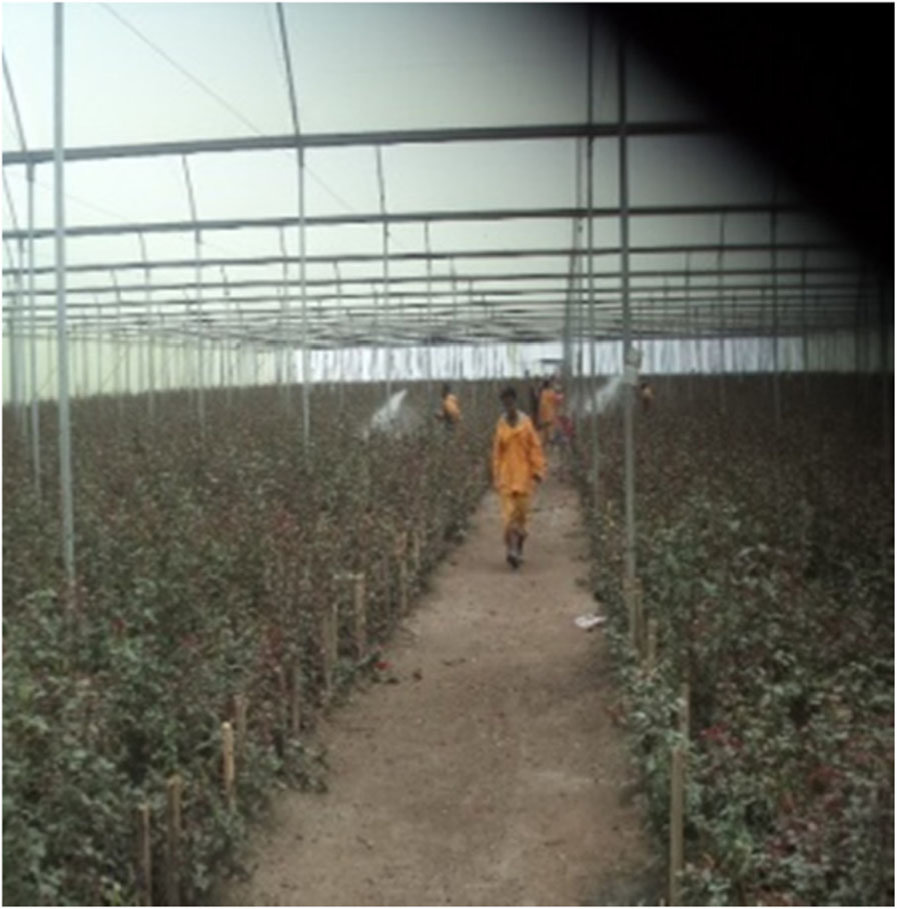
Workers’ practice of safety on duty

We observed majority of the workers did their daily jobs without using PPE. Of those workers who sprayed chemicals, only a few workers wear PPE, but they did not follow the direction of winds. The shortage offacilities such as drinking water, shower, toilets, and hand washing facilities were noticed in the firms. Therewere no warning signs at the entrance, particularly in the green house department. In the cold room department, both female and male workers had been working without the use of PPE. In many circumstances, chemical sprayers, supervisors and their assistants did not consider wind directions. The reasons mentioned were lack of awareness of how to work with chemicals, lack of PPE provision based on the importance and nature of activities and the limited supply of PPE itself made it difficult to replace when it was damaged by chemicals (Fig. 3).

### Factors associated with level of knowledge on occupational hazards

The bivariate linear regression analysis showed that there was statistically significant association between the knowledge of occupational hazards and sex, age, residence, type of employment, employment duration, level ofeducation status health and safety training and orientation, and safety instructions in local language. The multivariate regression analysis identified that level of education, type of employment and work experience were factors that considerably affected the level of knowledge on occupational hazards. The level of knowledge onoccupational hazards was more likely to increase by a factor of 20.03 for respondents who had diploma and higher education than those who had no formal education [AOR: 20.03; 95% CI (16.30, 23.75)]. Respondents with > 2 years of work experience were 5.97 times more likely to be knowledgeable about workplace hazards than those who had < 2 [AOR: 5.97: 95% CI (4.22, 7.72)]. Knowledge on occupational hazards was 2.34 times more likely to increase among participants who received health and safety trainings than those who did not [AOR: 2.34; 95% CI (1.73, 3.95)] (Table 4).

**Table 4:** Factors affecting the level of knowledge on occupational hazards (*N* = 451), 2017, Ethiopia

Characteristics	Number (%)	Knowledge of occupational hazards
		Un standardcoefficient for β (95% CI for β)
**Sex**		
Male	126 (27.9)	1.03 (−0.47, 2.54)
Female	326 (72.1)	1
**Age**	78 (17.3)	1
< 20 years		
20–29 years	170 (37.7)	−0.22 (−2.36,1.93)
≥ 30 years	203 (45.0	−0.47 (− 2.35,1.399)
**Educational status**		
No formal education	169 (37.2)	1
Primary (1-6grades)	117 (25.9)	2.37 (0.4,4.33)
Secondary (7–12 grades)	118 (26.2)	6.01 (2.95,9.07) **
Diploma and higher education	47 (10.42)	20.03 (16.30,23.75) **
**Residence**		
Urban	247 (54.8)	1.38 (−1.24,3.27)
Rural	204 (45.2)	1
**Types of employment**		
Temporary	211 (46.8)	1
Permanent	240 (53.2)	5.35 (2.50,8.19) **
**Work experience**		
< 2 years	210 (46.6)	1
≥ 2 years	241 (35.7)	5.97 (4.22,7.72) **
**Health and safety training**		
No	320 (71.0)	2.34 (1.73,3.95) *
Yes	131 (29.0)	1
**Safety instructions in local languages**		
No	51 (11.3)	2.9 (0.89,4.92)
Yes	400 (89.7)	1

**Keys:** 1 = reference category, ** = significant at *p* < 0.001, * = significant at *p* < 0.05 and R^2^Adjusted = 0.711 (shows model fitness; 71.1% variations are explained by variables in the model and 28.9% left as unexplained): *MSDs* Material safety data sheets

### Factors associated with level of safety practice

In the bivariate linear regression analysis, level of education, work experience, conditions of employment, health and safety trainings, presence of safety communications/signs, like symbols and labels in the local language, observance of work rules and regulations, regular communication and supervision on health and safety, use of material safety data sheets, provision of comfortable PPE, knowledge on occupational hazards and regular use of PPE were factors significantly associated with the level of safety practice.

In the multivariate linear regression analysis, the provision of comfortable PPE, regular use and storage of PPE, presence of safety communications/signs, and knowledge on workplace hazards markedly influenced workers’ level of safety practice. The provision of PPE increased the levels of safety practice 4.59 times more likely compared to no provision of it [AOR: 4.59; 95% CI (2.34, 6.86)]. The level of safety practice was 5.15 times higher for respondents who worked where health and safety communications available than in the absence of such communications [AOR: 5.15; 95% CI (1.81, 8.49)]. The level of safety practice was 7.29 times higher for participants who had knowledge on occupational hazards than those who had not [AOR: 7.29; 95% CI (3.87, 10.73)] (Table 5).

**Table 5:** Factors associated with the level of safety practice (*N* = 451), 2017, Ethiopia

	Frequency (%)	Safety practice
		Unstandard coefficient for β (95% CI for β)
**Educational level**		
No formal education	169 (37.2)	1
Primary (1–6 grades)	117 (25.9)	2.22 (−0.29,4.73)
Secondary (7–12 grades)	118 (26.2)	1.32 (−2.73,5.47)
Diploma and higher	47 (10.7)	1.09 (−3.92,6.12)
**Work experience**		
< 2 year	210 (46.6)	1
≥ 2 years	241 (53.4)	−1.53 (−3.84,0.78)
**Types of employment**		
Temporary	211 (46.8)	1
Permanent	240 (53.2)	1.10 (−2.61,4.81)
**Health and safety training**		
No	320 (71.0)	−0.32 (−2.41,1.77)
Yes	131 (29.0)	1
**Safety communications/signs at work**		
No	400 (88.7)	1
Yes	51 (11.3)	5.15 (1.81,8.49) **
**Provision of comfortable PPE**		
No	74 (16.4)	4.59 (2.34,6.86) **
Yes	377 (83.6)	1
**Use of material safety data sheets (MSDS)**		
No	286 (63.4)	1
Yes	165 (36.6)	−0.38 (−2.37,1.61)
**Regular use of PPE**		
No	330 (73.2)	1
Yes	121 (26.8)	17.53 (13.36,21.71) **
**Observe work rules and regulations**		
No	237 (52.3)	1
Yes	214 (47.5)	0.13 (−1.80,2.06)
**Presence of supportive supervision and inspection**		
No	416 (92.2)	1
Yes	35 (7.8)	0.34 (−3.01,3.69)
**Knowledge of occupational hazards**		
No	274 (60.8)	1
Yes	177 (39.2)	7.29 (3.87,10.73) **
Constant		34.054 (25.54,42.57) **

**Keys:** 1 = reference, ** = significant at *p* < 0.001, * = significant at *p* < 0.05 and R^2^Adjusted = 0.683(Shows model fitness (68.3% explained variations by variables in the model and 31.7%Unexplained left)

## Discussion

Recognizing the health and safety barriers and facilitators is a key to the successful implementation of health and safety programs. In this cross-sectional survey, we assessed the knowledge and practice of occupational hazards and their associated factors among flower farm workers in Southwest Shewa zone, National Regional State of Oromia, Ethiopia. The finding of this study shows that the levels of knowledge and safety practice were 39.2% (*N* = 177) [95% CI (34.8, 43.9)] and 26.6% (*N* = 120) [95% CI (22.6, 30.6)], respectively**.** Our results suggest that more than half of the participants (60.8%) were not knowledgeable and the majority (73.4%) had poor preventive practice of workplace hazards. This could be because in Ethiopia, despite the inaugurations of few promising initiatives on health and safety enforcement, and its coverage since the past decade, the practical implementation yet remains indescribable.

In this investigation, the level of knowledge on occupational hazards was comparable to that of a study report in Palestine (42%) [27]. This similarity may be because the implementation of health and safety programs such as health and safety training, compliance with available safety standards and regulations, and health and safety policy development are generally substandard in developing countries. The result was, however, lower compared to those of studies conducted in Ethiopia (72%) [28], India (70%) [29] and Mexico (50%) [30]. The possible reason could be because of differences in methods of data collection and study populations. Other possible explanations might be because of differences in access to health and safety services, available regulations on safety and health enforcement, and workplace safety culture.

In the current study, the level of safety practice was 26.6% (*N* = 120) [95% CI (22.6, 30.6)]. This result was lower compared to that of a study conducted in Jamaica (36.7%) [31], China (32.3%) [32], Brazil (80%) [23], India (60%) [29], the Philippines (91%) [33], Palestine (63.5%) [27], and the Amazon Basin (99.1%) [34]. Possible suggestions for these differences might be because of variations in access to policies on health and safety regulations and standards at farm level as well as socio-economic and cultural distinctions across countries.

Our analysis demonstrated that the level of knowledge of workerson occupational hazards was significantly influenced by the level of education. This result was supported by studies conducted in Ethiopia [15], Tanzania [22, 35], Kenya [36], Palestine [27], and Nepal [37]. The possible explanation could be that education could improve understanding of workers and foster a culture of safety in the workplace. Further, education can make life easier and boosts a means of communication for acquiring knowledge. The results from the studies conducted in Tanzania [22], Jamaica [31], and India [38] were inconsistent with our finding. This might be due to differences in socio-economic characteristics of the workers, data collection techniques, and sample sizes.

In the current analysis, the length of employment/work experience was considerably related to the level of knowledge of the participants. This result confirms the studies in Palestine [27], Nepal [37], and the Amazon Basin, Ecuador [34] but disagrees with that of a study in India [38]. The length of employment can increase exposure of workers to a variety of workplace hazards. This, in turn elevates the awareness, anticipation, and recognition of workers about hazards over the years.

We found the type of employment/permanent versus temporary/noticeably affected the knowledge of employees on workplace hazards. Similar findings have been documented in Palestine [27] and India [38]. A possible explanation is that permanent workers usually hold a good occupational status and are more likely to participate in trainings and other skill advancement opportunities. Another possible reason might be that workers with permanent contracts of employment are usually well trained with relatively high payments, which in turn enhance knowledge seeking behavior of workers.

Safety training was the other factor that considerably affected the knowledge of employees on workplace hazards. The findings in Brazil [24] and Jamaica [31] were in line with this result. The probable explanation may be that health and safety training is an important resource to improve anddevelop the capacity of workers to tackle risks associated with their careers. Our analysis, however, was discordant with that of a report in China [39] and the Philippines [33]. The possible reasons for these variations may be because of differences in access to health and safety trainings, the availability of agricultural-specific labor standards and regulations, sample sizes, study populations, methods of data collection, and analysis.

The lack of written health and safety instructions (for example, labels, symbols, pictograms) in the local languages showed a significant relation with the knowledge of workers. This was supported by the studies in Ethiopia [15], Jamaica [31], and Lesotho [21]. The possible explanation is that workers can easily understand and comply with the available safety instructions and guidelines without restrictions if those instructions and guidelines are presented in the native language of the workers at work. In Ethiopia, one can appreciate a diverse ethnic group with different languages in a particular workplace. While the national language is also available for work, the majority of essential safety communications and signs at the workplaces are often presented with the languages of those foreign investors. Employers and concerned officials in Ethiopia need to basically look at for this gap. However, our finding is contradictory to the literature in Tanzania [22] may be because of discrepancies in sampling procedures and study populations.

In this study, regular use and safe storage of PPE was significantly associated with safety practice. There have been concurrent findings from recent works in Tanzania [22], Palestine [27], and India [38]. Moreover, the knowledge of workers positively influenced level of safety practice. This finding agreed with those of reports in Palestine [27] and India [29]. Knowledge of workers on occupational hazards in a particular workplace would possibly strengthen the control mechanisms of those hazards.

In the current report, the result of multivariable linear regression analysis unveiled that safety communications importantly affected the level of safety practice. The reliable findings were documented in the studies in Zimbabwe [7] and Palestine [27]. This may explain that the availability of various safety communications at work would likely prompt employees to observe safety rules and regulations in their daily routines. Moreover, the provision of appropriate PPE was significantly associated with the level of safety practice. Comparable findings have been published in Zimbabwe [7], Tanzania [22], and Palestine [27].

The data used in this analysis werebased on self-reports of the workers. Therefore, constraints because ofa recall bias cannot be avoided. The recent (3 months) experiences of the workers have been gathered to reduce the effects. Second, due to the expediency of the authors, data on the attitude of the workers on occupational hazards were not obtained, which indeed, was not the aim of the study. However, we conclude that sufficient attention is paid to the most important elements of occupational hazards including knowledge and practice, which provide a better indicator for program implementer. Moreover, it might be difficult to explore the level of knowledge using quantitative data alone. Therefore, the other shortcomings of this analysis may be the lack of qualitatively triangulated data to investigate, for example, why the workers were not using PPE properly. Future investigations, therefore, had better focus on better data collection techniques, such as qualitative methods to explore knowledge related factors that influence safety practice of workers.

## Conclusion

The study revealed the levels of knowledge and practice of occupational hazards were low. Knowledge on occupational hazards was significantly affected bythe level of education and duration of employment. Moreover, the use of PPE and level of knowledge considerably influencedsafety practice. Therefore, we recommend employers to ensure that workplace health and safety programs need to account for workers’ level of education and their work experience. It is also pivotal to provide workers witha suitable PPE and instructions on its use, and to arrange safety communication in the local language at relevant workplaces.

## Supplementary Information

**Additional file 1.** The survey questionnaire employed for data collection.

## Data Availability

Authors presented the data in the main paper and made available from the corresponding author on reasonable request.

## Abbreviations

AOR: Adjusted odds ratio
CI: Confidence interval
CI: Confidence interval
EOHS: Environmental and Occupational Health and Safety
ILO: International labor organization
Km: Kilometers
MPH: Masters of public Health
MSc: Masters of Science
MSDS: Materialsafety data sheets
OR: Odds ratio
PPE: Personal protective equipment
SD: Standard deviation
SPSS: Statistical package for social sciences
USD: United States of America Dollar
