# Supplementary material for: Low levels of knowledge and practice of occupational hazards among flower farm workers in southwest Shewa zone, Ethiopia: a cross-sectional analysis
Source: BMC Public Health. 2021 Jan 28;21:232. doi: 10.1186/s12889-021-10254-5 (PMC7844994; doi:10.1186/s12889-021-10254-5)
Supplement: Supplementary file 1 — Additional file 1. The survey questionnaire employed for data collection. [file 12889_2021_10254_MOESM1_ESM.docx]

## 9.2. Annex 2: English questionnaires

University of Gondar, College of Medicine and Health Science,

Institute of Public Health.

Questionnaire for the Knowledge, Practice and Associated factors of Occupational Hazards among Floriculture Workers of Woliso, Ethiopia, 2015.

Company name/office………………………………

Working department………………………..

Questionnaire identification number……………

Hello! My name is ………………………………….. I am here on behalf of Debela Hinsermu, student of master of public health at institute of public health, University of Gondar. He is conducting research for partial fulfillment of master degree on Knowledge, practice and associated factors of occupational hazards among floriculture workers of Woliso. He has permission from University of Gondar. Like you, Workers from production are randomly selected to participate in this study. We are kindly requesting you to answer question that we have prepare for you and those study questionnaire will take maximum of 20 minute. We assure that all information gathered during the course of study will be kept strictly confidential and the information is used for this research only. All the information you going to give us will be coded for anonymity. Only the principal investigator and data collector will have access to the data.

Are you willing to participate in the study? 1. Yes 2. No

If you say “yes” sign below…. thank you so much

Signature ___________________ Date _________________

If no, skip to the next participant by writing the reason of his/her refusal.

Informed consent certified by:

Interviewer code_______ Name __________________ signature_________

Date of interview________ time started __________time completed ______

Result of interview:

1 complete 2 refused 3 respondent not available 4 partially completed.

Checked by: Supervisor name __________ signature ________date ________

**Part i: - Socio-demographic questions**

| S/N | Question | Response | Code |
| --- | --- | --- | --- |
| 101 | Identification number | ___________ |  |
| \| 1102 \|  \|  \| \| --- \| --- \| --- \| \|  \| \| \| | Sex: | 0 = male |  |
|  |  | 1 = female |  |
| 103 | Age | ………………year |  |
| 104 | Ethnicity | ………………….. |  |
| 105 | Marital status | 1= single |  |
|  |  | 2 = married |  |
|  |  | 3 = widow |  |
|  |  | 4 = separated/divorced  5=living with friend |  |
| 106 | Education status | 1= No formal education |  |
|  |  | 2 = primary school |  |
|  |  | 3 = secondary school |  |
|  |  | 4 = above |  |
| 107 | Working department | ……… department |  |
|  | Work position in the department | ------------------------------ |  |
| 108 | How long on this job or work experience? | ………months/years |  |
| 110 | How many working hours per day? | ..................hrs/day |  |
| 111 | How many working hours per week? | .................hrs/week |  |
| 112 | State working shifts | ---------------- |  |
| 113 | What is your salary per month? | ……………Eth.Birr |  |
| 114 | Conditions of employments | 1= temporary  2= permanent |  |
| 115 | General conditions of the department | 1= ventilated  2=overcrowded |  |

**Part II Knowledge Questions**

**i)Related to Information about Occupational hazards**

| **Sr. no** | **Question** | **Response** | **Code** |
| --- | --- | --- | --- |
| 201 | Have you ever heard about Occupational health hazards? | 0.No  1.Yes |  |
| 202 | Did you know the occupational hazards associated with their current jobs | 0.No  1.Yes |  |
| 203 | Have you been informed the about the nature and working conditions of working processes? | 0.No  1.Yes |  |
| 204 | What is your source of information about these workplace hazards?(two or more than two answer is possible) | 1.training given by company  2. from guideline given by manufacturer  3.from experienced workers  4.From media | |
| 205 | Do you have awareness about the forms in which workplace hazards can exist? | 0.No  1.Yes | If yes state forms |
| 206 | Do you know any law regarding Occupational Health and Safety in the flower farm? | 0.No  1.Yes | If yes,what is it about? |
| 207 | Do you have an aware and understand the health and safety instructions(Danger sign, warnings and Cautions) |  |  |
| 208 | Do you have an understanding of six types of work place hazard that may you face? | 0.No  1.Yes | **If no**  **Q.209** |
| 208.1 | Chemical hazards | 0.No  1.Yes |  |
| 208.2 | Biological hazards | 0.No  1.Yes |  |
| 208.3 | Physical hazards | 0.No  1.Yes |  |
| 208.4 | Psychosocial hazard | 0.No  1.Yes |  |
| 208.5 | Ergonomical hazards | 0.No  1.Yes |  |
| 208.6 | Mechanical hazards | 0.No  1.Yes |  |

**ii) Questions of knowledge related to routes of exposure, Health problems and preventive measures of occupational hazards.**

| **Sr. no** | **Question** | **Response** | **Code** |
| --- | --- | --- | --- |
| 209 | Do you have information of work place hazards routes of exposure? | 0.No  1.Yes | **If no**  **Q.210** |
| 210 | Can differentiate the major routes of exposures? | 0.No  1.Yes | If yes, state each |
| 211 | Did you know the possible health problems arise from the existing hazards? | 0.No  1.Yes | If yes, specify |
| 212 | Do you have an information about workplace hazards can cause environmental pollutions? | 0.No  1.Yes | If yes, mentioned it. |
| 213 | Do you know your rights and responsibilities in accident reduction/prevention strategy? | 0.No  1.Yes | If yes, state each |
| 214 | Do you aware of about emergency response and preparedness for the control of work place hazards | 0.No  1.Yes | If yes, state each |
| 215 | Do you know the use of emergency measures in the time of accidence occurrence? | 0.No  1.Yes | If yes, state use |

**Questions related to Practices**

| **Sr. no** | **Question** | **Response** | **Code** |
| --- | --- | --- | --- |
| 301 | Do you follow safe procedure (following safety rule, instruction, warning sing and alarm) and safety measures to reduce dangerous occurrence at work? | 0.No  1.Yes |  |
| 302 | Have you been practiced safe procedures of to control occurrence accidents | 0.No  1.Yes | **If yes state** |
| 303 | Do you using the personal protective equipment while working? | 0.No  1.Yes | **If no. Q.307** |
| 304 | Are you satisfied with protective equipment provided? | 0.No  1.Yes | **If no,why** |
| 305 | Have you been followed advise/instruction to the use of protective equipment? | 0.No  1.Yes |  |
| 306 | Among these PPE Which are you using carefully from the following? |  |  |
|  | 306.1. Wear an eye mask? | 0.No  1.Yes |  |
|  | 306.2. Wear special gloves? | 0.No  1.Yes |  |
|  | 306.3. Wear special shoes? | 0.No  1.Yes |  |
|  | 306.4. Wear special clothes? | 0.No  1.Yes |  |
|  | 306.5. Wear a special face mask? | 0.No  1.Yes |  |
|  | 306.6.Over all | 0.No  1.Yes |  |
|  | **Questions of practices related to safe and unsafe behaviors** | | |
| 307 | Do you follow the instruction of chemical and pesticides bottle's label given by MSDS? | 0.No  1.Yes |  |
| 308 | Do you have safe disposal of the empty containers of chemicals, pesticide and fertilizers? | 0.No  1.Yes | **If No**  **Q.310** |
| 309 | Among these, which mechanism have you been practiced? |  |  |
|  | Store in separate area | 0.No  1.Yes |  |
|  | Burning | 0.No  1.Yes |  |
|  | Burying | 0.No  1.Yes |  |
|  | Washing and reusing at home | 0.No  1.Yes |  |
|  | Reuse for storage of other pesticide | 0.No  1.Yes |  |
| 310 | Have you been used empty containers of chemicals, pesticides and fertilizers for uses? | 0.No  1.Yes | **If No Q.311** |
|  | 310.1.For storage of water and other raw materials | 0.No  1.Yes |  |
|  | 310.2.For selling purpose | 0.No  1.Yes |  |
| 311 | Have you never take part unrecompensed activities during working in hazardous areas of production flow? | 0.No  1.Yes | **If No Q.312** |
|  | 311.1.Do you eat during working | 0.No  1.Yes |  |
|  | 311.2. Do you drink during working | 0.No  1.Yes |  |
|  | 311.3. Do you smoke during working | 0.No  1.Yes |  |
|  | 311.4. Not Washes hands after work and before eating | 0.No  1.Yes |  |
|  | 311.5. Not using Separates clothes when washing | 0.No  1.Yes |  |
|  | 311.6. Not Reads labels on pesticide containers | 0.No  1.Yes |  |
|  | 311.7. taking PPE to home and washing at home | 0.No  1.Yes |  |
| 312 | Do you use properly first aid service available in workplace when you may affected by hazards? | 0.No  1.Yes |  |
| 313 | Do you follow proper communication between you and your coworkers | 0.No  1.Yes |  |
| 314 | Have you been used manufacturers providing material safety data sheets (MSDS) properly? | 0.No  1.Yes | **If yes, specify.** |
| 315 | Do you use information provided by manufacturers properly for reduction of workplace hazards? | 0.No  1.Yes |  |
| 316 | Have you ever had safe handling of work processes and materials? | 0.No  1.Yes | **If yes, specify** |
| 317 | When spraying chemicals and pesticides do use & follow wind directions? | 0.No  1.Yes | **“** |
| 318 | Are you experienced in immediate reporting of sudden occurrences? | 0.No  1.Yes | **“** |

**Organizational and personal (behavioral factors that affecting knowledge and practices**

| **Sr. no** | **Question related to organizational factors** | **Response** | **Code** |
| --- | --- | --- | --- |
| 401 | Have you received specialized health and safety training on the existing hazards? | 0.No  1.Yes | If yes,  State it. |
| 402 | Have you motivated in promotion of health and safety participation | 0.No  1.Yes |  |
| 403 | Did you obtain management support and orientation on safety signs | 0.No  1.Yes |  |
| 404 | Have got regular supervision and communication of health and safety | 0.No  1.Yes |  |
| 405 | Have you been awared the Farm health and safety policy? | 0.No  1.yes |  |
| 406 | Are there available instructions of health and safety in local language | 0.no  1.Yes |  |
| 407 | Are the provision of adequate welfare sanitary facilities in your farm | 0.No  1.Yes |  |
| 408 | Is there availability of health and safety posters, labels | 0.No  1.Yes |  |
| 409 | Are there provisions of comfortable PPE to wear?  (considering natures & environmental conditions) | 0.No  1.Yes | If no ,state reasons |
| **Factors related to behaviors/ personal** | | | |
| 410 | Do you use Material safety data sheets for raw materials that provided at work? | 0.No  1.Yes |  |
| 411 | Are you awared and exercising your right and obligation well? | 0.No  1.Yes |  |
| 412 | Do you taking some stimulants?  If yes ,Mention the frequency of uses | 0.No  1.Yes | If yes ,state types |
| 412 | Regular use of PPE and storing in designed area after use | 0.No  1.Yes |  |
| 413 | Are there easy observable instructions fixed on raw materials used at work? | 0.No  1.Yes | **If no,Pls specify.** |
